# Cannabis use and beliefs among people in recovery from substance use disorders

**DOI:** 10.1186/s42238-026-00461-5

**Published:** 2026-07-03

**Authors:** Anthony N. Nist, Christine E. Sheffer, Ghada Nusair, Allison N. Tegge

**Affiliations:** 1 Fralin Biomedical Research Institute at Virginia Tech Carilion, Roanoke, VA 24016 USA; 2 Department of Health Behavior, Roswell Park Comprehensive Cancer Center, Buffalo, NY 14203 USA

**Keywords:** Cannabis, SUD recovery, Beliefs, Acceptability, Safety, Risks, Benefits, Addictiveness

## Abstract

**Background:**

Cannabis use has increased dramatically in the past decade and is currently among the most commonly used drugs in the world. Among people in recovery from substance use disorders (SUDs), cannabis is sometimes put forward as a less harmful substitute for other drugs, conflicting with abstinence-based recovery approaches. We aimed to describe cannabis use among people in recovery, explore relations among cannabis use (CU) status (Current-CU, Former-CU, Never-CU) and beliefs about social acceptability, safety, benefits, risks, and addictiveness of cannabis, and compare beliefs about the addictiveness of cannabis with that of other substances.

**Methods:**

This study was a cross-sectional, online survey of people in recovery (*n* = 507) from SUDs. Cannabis use and beliefs about acceptability, benefits, risks, safety, and addictiveness were assessed, and diagnostic criteria for SUDs, including cannabis use disorder (CUD).

**Results:**

Participants were, on average, 47.2 (SD = 12.2) years old, 67.3% female, 81.1% White, and 93.7% non-Hispanic/Latino; 35.9% and 51.3% indicated Current- or Former-CU, respectively. Current-CU was associated with use for therapeutic purposes, and more positive beliefs about the acceptability (*p*s ≤ .010), safety (*p*s < .001), and benefits of cannabis, and risks compared to Former- or Never-CU. Those who never used cannabis rated cannabis as the least socially acceptable (*p*s ≤ .015), least safe (*p*s ≤ .036), and were least likely to endorse potential benefits. Individuals who formerly used cannabis generally showed intermediate beliefs. All participants believed cannabis was less addictive than alcohol (*p*s < .001), cigarettes (*p*s < .001), and cocaine (*p*s < .001), but Current-CU believed cannabis was significantly less addictive (*p*s < .001) than Former- and Never-CU. Participants with mild CUD believed cannabis was less addictive than those with severe CUD. Among participants with other SUDs, beliefs about the addictiveness of the respective substance did not differ by SUD severity (*p*s ≥ .298).

**Conclusions:**

These findings underscore the importance of recognizing heterogeneity in cannabis use among people in recovery. Those with mild CUD believed cannabis was the least addictive while those with severe CUD believed cannabis was most addictive. In contrast, beliefs about the addictiveness of other substances were independent of SUD severity. This pattern of beliefs about addictiveness appears to be specific to cannabis. Understanding beliefs about cannabis use among people in recovery may clarify its impact on recovery.

**Supplementary Information:**

The online version contains supplementary material available at https://doi.org/10.1186/s42238-026-00461-5.

## Background

Cannabis is the third most commonly used drug in the US and the world, after alcohol and tobacco. (Karila et al. 2014; Connor et al. 2021; Abuse 2019) Driven by legalization of cannabis use, an explosion in the variety of cannabis products, and expanded therapeutic use, there has been a 70% increase in past-month cannabis use among adults in the US in the past decade. (World Drug Report 2021; Martins et al. 2021; Han et al. 2021; Yu et al. 2020) Among young adults, past month and daily cannabis use has nearly doubled in that timeframe, while in 2021, 11% of young adults reported daily cannabis use, the highest prevalence ever recorded. (NIDA 2021) At present, the majority of Americans have legal access to cannabis, and about 48.2 million people, 18% of Americans, use cannabis at least once a year. (Centers for Disease Control and Prevention 2021) This evolving landscape has also had a significant impact on cannabis use among people in recovery from substance use disorders (SUDs).

Beliefs about cannabis use are important to understand the sociocultural climate around cannabis use in different populations. (Florimbio et al. 2024; Grevenstein et al. 2015; Doggett et al. 2025) With the dramatic increases in cannabis use and availability, these beliefs about cannabis use including social acceptability, safety, benefits, risks, and addictiveness have evolved, but not always in the expected directions. (Doggett et al. 2025; Wen et al. 2019; Mennis et al. 2023; Carliner et al. 2017) For instance, legalization in Canada has been associated with increases in the acceptability of recreational cannabis use, but was also followed by heightened awareness of risks, increased safety concerns including concern about addictiveness, and decreases in positive beliefs about the therapeutic benefits of use. (Doggett et al. 2025) In the US, however, following recreational legalization, a large national survey found that the associations between higher cannabis use prevalence rates and lower perceived risk of use were strengthened. (Mennis et al. 2023) In both Canada and the US post-legalization, frequent cannabis users demonstrate substantial deficits in knowledge about the health risks of cannabis. (Wen et al. 2019; Goodman and Hammond 2022) These findings highlight the need to monitor beliefs about cannabis as cannabis legalization policies evolve, especially among high-risk groups like people in recovery from SUDs.

About 1 in 10 (23.5 million) adults in the US consider themselves in recovery from SUDs. (Key Substance Use and Mental Health Indicators in the United States 2024) Among these adults, about 90% have ever used cannabis, and about one-third have used cannabis in the past month. (Jones et al. 2020) Traditional recovery approaches require abstinence from all substances, except tobacco. (Kaskutas et al. 2014) Although, as the definition of recovery has evolved to focus on improvements in functioning, total abstinence requirements are being questioned. (Witkiewitz et al. 2020; Paquette et al. 2022) Further, cannabis is being proposed as a substitute for alcohol, stimulants, opioids and other substances (Reiman et al. 2017; Reiman 2009; Subbaraman et al. 2025; Beaugard et al. 2024; Mikuriya 2004) as well as a method for treating anxiety, depression, and other mental health disorders among people in recovery. (Leung et al. 2022) However, a recent systematic review found insufficient evidence for the safety and efficacy of using cannabis for the treatment of SUDs as well as the treatment of anxiety, post-traumatic stress disorder, ADHD, bipolar disorder, and obsessive–compulsive disorder among people with SUDs. (Wilson et al. 2026) Although many studies show no relation between cannabis use and SUD treatment and/or recovery outcomes (Wilson et al. 2026), several studies have found cannabis use to have a negative impact on treatment and recovery. (Franklyn et al. 2017; Lake and St Pierre 2020; Mojarrad et al. 2014; Aharonovich et al. 2005; Weinberger et al. 2016) For example, cannabinoid use has been associated with decreased treatment retention among people with opioid use disorder (Franklyn et al. 2017), lower abstinence rates among people who use alcohol and other substances (Mojarrad et al. 2014), an increase in cocaine craving among those with cocaine use disorder (Gallassi et al. 2025; Meneses-Gaya et al. 2021; Mongeau-Pérusse et al. 2021), and negative impacts on remission among people with alcohol use disorder. (Subbaraman et al. 2025; Aharonovich et al. 2005) Together, this suggests a significant disconnect between the therapeutic use of cannabis and research findings among people in recovery from SUDs.

Given these factors, cannabis use is likely to have distinctive meaning and ramifications for people in recovery. For instance, people in recovery might have an enhanced awareness of the addictiveness of all substances including cannabis, or might view cannabis as a lower-risk alternative or therapeutic substitute for other substances. (Lau et al. 2015; Fehr et al. 2024; Mok et al. 2023; Swartz 2010; Warnock et al. 2024) People in recovery may have had negative experiences with cannabis that shape more negative perceptions of cannabis, (Ellingstad et al. 2006; Hodgins and Stea 2018) consistent with evidence that those who seek treatment for cannabis use disorder (CUD) often attribute multiple problems to cannabis use such as memory problems, sleep disruptions, and issues with personal relationships. (Budney et al. 1998; Copersino et al. 2006; Hughes et al. 2008; Stephens et al. 2002) Qualitatively, people in recovery have described positive beliefs about cannabis’s therapeutic benefits, and some believe that when used alone it is largely harmless. In contrast, others believe that cannabis was a gateway to their problems with addiction and is an issue in their recovery, and thus may hide their cannabis use due to negative peer perceptions and interference with treatment-related activities. (Warnock et al. 2024) Therefore, understanding the perceptions of social acceptability, safety, benefits, risks, and addictiveness of cannabis is vital for understanding the role of cannabis in addiction treatment models and tailoring individualized treatment plans.

This study’s objectives were to 1) describe cannabis use among a large convenience sample of people in recovery from common SUDs, and among this sample 2) explore beliefs about social acceptability, safety, benefits, risks, and addictiveness of cannabis among participants who currently, formerly, or never used cannabis use, and finally, 3) compare beliefs about the addictiveness of cannabis with that of other substances accounting for the level of severity of each SUD. Based on a recent study (Jones et al. 2020), we hypothesized that most participants (~ 90%) would have experience using cannabis, participants who currently use cannabis would have more positive beliefs about cannabis use than those who formerly or never used cannabis, and the addictiveness of cannabis is less than other substances.

## Methods

### Participants and procedure

In this study, we examined cannabis use and beliefs in a large cohort of people in recovery. Participants were members of the International Quit and Recovery Registry (IQRR; https://www.quitandrecovery.org/), an ongoing project sponsored by the Fralin Biomedical Research Institute at Virginia Tech Carilion. The IQRR provides a platform where registrants from anywhere in the world can connect with recovery resources as well as participate in research. Participants typically learn about the IQRR platform through study recruitment efforts such as flyers, social media, recovery community organizations, treatment facilities, and referrals from family and friends. Some participants also report learning about the IQRR through Google and other search engines. Registrants (*n* = ~ 13,000 at present) have varying levels of engagement in IQRR community and research activities. In order to be eligible to participate in monthly research studies individuals must: 1) be 18 years or older; 2) self-identify as being in recovery from at least one SUD where recovery is defined as “making active changes to cut down or eliminate substance use, regardless of successe.g., (Fontes et al. 2025; Nist et al. 2025); and 3) meet or have met Diagnostic and Statistical Manual-5 (DSM-5) criteria for at least one SUD (American Psychiatric Association 2013) during their lifetime (note cannabis use was not an inclusion criteria). The data for this study was cross-sectional and collected via a single online survey (Qualtrics, Provo, UT) administered to IQRR registrants in July 2025. Participants received a link to the survey via email or could access the survey by logging into the IQRR website. To prevent non-registrants from accessing the survey, only eligible registrants with verified email addresses could access the survey. No other inclusion criteria for this survey were assessed beyond that described above. Participation was voluntary and consent was inferred from the completion and submission of the survey. The Institutional Review Board at Virginia Polytechnic Institute and State University approved and monitored this study and the overall IQRR. IQRR participants are provided escalating compensation for completing monthly surveys with a minimum of $5, a maximum of $7, and a bonus of $5 for every three surveys completed.

### Measures

Participants provided sociodemographic information including age, sex, race, ethnicity, years of education, household income, legal status of cannabis where they reside, and details about past and current use of substances.

#### Cannabis use status

Participants were grouped based on their self-reported history of cannabis use: never used cannabis (Never-CU), formerly used cannabis (Former-CU), and currently use cannabis (Current-CU).

#### Substance use history

In this survey, participants were asked which of 11 substances they had used problematically in their lifetime. For each substance endorsed, they completed a symptom checklist that assessed DSM-5 SUD criteria experienced in the past 3-months. For this study, we included only those participants who endorsed past three month criteria for one of alcohol use disorder (AUD), CUD, cocaine use disorder (CoUD), and/or tobacco use disorder (TUD), the most frequently endorsed SUDs in the IQRR. (Fontes et al. 2025; Nist et al. 2025; Cabral et al. 2024) Unlike an in-depth clinical assessment, the self-report symptom checklists did not assess clinical impairment or distress. Symptom checklists have been validated against clinically administered assessments of SUDs and have shown excellent test–retest reliability. (Hallgren et al. 2024; Hasin et al. 2020; Matson et al. 2023) Specifically, participants answered 11 questions related to specific symptoms (i.e., craving, tolerance, withdrawal, etc.) which were then summed to provide a total score. Consistent with the diagnostic literature, a score of 0–1 symptoms was categorized as No SUD, 2–3 symptoms Mild SUD, 4–5 symptoms Moderate SUD, and 6 + symptoms Severe SUD. (Hasin et al. 2013) Individuals that did not endorse problematic use of a substance were considered to have ‘never used’ the substance.

#### Cannabis use characteristics

All participants were asked”How many of your close friends or family members use cannabis (either recreationally or medically?” and “How often are you around people who are using cannabis?”. In addition, participants were asked “Where you live currently, what is the legal status of cannabis right now?” and “In your opinion, what should the legal status of cannabis be?”. Finally, participants were asked to indicate how knowledgeable they thought they were about cannabis products and their effects. Knowledge was assessed on a 5-point discrete analogue scale ranging from “Not at All” to “Extremely.” Only participants who reported current or former cannabis use were asked the age at which they first tried cannabis, how often they currently use or formerly used cannabis, and if they use/used cannabis for therapeutic purposes.

### Beliefs about cannabis

#### Social acceptability

Consistent with the wording used in previous studies (Doggett et al. 2025; Salas-Wright et al. 2021), participants were asked “How socially acceptable do you think it is for a person to…?” 1) Use alcohol regularly, 2) Smoke cigarettes regularly, 3) Use cannabis regularly if it is for non-medical or recreational purposes, 4) Try cannabis once or twice for recreational purposes. Response options included “Completely unacceptable,” “Somewhat unacceptable,” “No opinion,” “Somewhat acceptable,” and “Completely acceptable.” Note regular use was left to interpretation by the participant.

#### Safety

Consistent with previous studies (Doggett et al. 2025; Keyhani et al. 2018; Lipari & Jean-Francois 2016), participants were asked “How much do people risk harming themself physically and in other ways when they use cannabis…” for each of the following four timeframes: 1) Occasionally (monthly or less), 2) Infrequently (2–4 times per month), 3) Frequently (1–3 times per month), and 4) Daily or almost daily (4 + times per week). Response options for each timeframe included “No Risk,” “Slight risk,” “Moderate risk,” and “Great risk.” Participants were then asked which of the previous options was “most safe” and “least safe.” Safety of cannabis use among pregnant women was assessed on a 4-point scale “Completely unsafe,” “Somewhat unsafe,” “Somewhat safe,” “Completely safe.” Finally, participants were asked to compare the safety of driving under the influence of cannabis with driving under the influence of alcohol. Response options included “Cannabis is much less safe,” “Cannabis is somewhat less safe,” “Cannabis and alcohol are equally safe,” “Cannabis is somewhat more safe,” “Cannabis is much safer.”

#### Benefits and risks

Consistent with previous studies (Doggett et al. 2025; Keyhani et al. 2018), participants were provided with lists of potential benefits and risks of using cannabis. Participants were asked to select all the benefits and risks of using cannabis. The benefits included Pain management; Treatment of disease (such as epilepsy or multiple sclerosis); Relief from stress, anxiety, or depression; Improved appetite; Improved sleep; Help decreasing or stopping other medicines; Improved creativity; Improved focus or concentration; Increased Energy; Other; There are no benefits of using cannabis; I don't know. The risks included Legal problems; Addiction; Impaired memory; Increased use of other substances; Personal or relationship problems; Decrease in intelligence; Decrease in energy; New or worsening health problems; Increase in stress, anxiety, or depression; Disrupted sleep; Other; There are no Risks; I don’t know.

#### Addictiveness

The addictiveness of 5 substances (cannabis, opioids, tobacco/cigarettes, cocaine, and alcohol) was assessed on a 5-point scale ranging from “Not at All” to “Extremely,” regardless of prior history of each substance. The order in which the substances were presented was randomized.

### Statistical analysis

The IQRR invites all registrants to complete assessments, and thus does not enforce a cap on the sample size. As a result, an a priori sample size was not estimated. Given the observed sample size of *n* = 507, we performed a post hoc estimation of the smallest effect size of interest. Using three groups, alpha 0.05, 95% power, we are appropriately powered to uncover effect sizes as small as Cohen’s f = 0.175 (small-medium; continuous measures) and Odds Ratio = 2.68 (OR; binary measures). This analysis was not pre-registered and should be considered exploratory in nature.

Demographic and cannabis use characteristics are summarized using mean (standard deviation [SD]) and frequency (percentage), where appropriate. Demographics and cannabis use characteristics were compared among cannabis use status (current, former, never) using analysis of variance (ANOVA; for continuous measures) and Chi-square tests (for categorical measures). Cannabis use status was used to predict social acceptability, safety, risks, and benefits, in a series of logistic regression models. All non-binary outcomes (e.g., social acceptability, safety) were modeled as ordinal variables using proportional odds logistic regression with a cumulative logit link via the MASS package in R. (Venables and Ripley 2013) For binary outcomes (e.g., risks and benefits; 1 if endorsed, 0 if not) we used logistic regression models with a logit link. In all models, the “never” cannabis use status category was used as the reference variable and demographic covariates (age, sex, race, ethnicity, household income) were included as fixed effects.

In secondary analyses, we explored how beliefs about the addictiveness of cannabis differed from that of other substances by cannabis use status (current, former, never). We employed a linear mixed-effects model to examine associations among cannabis use status and the addictiveness of cannabis, alcohol, cigarettes, cocaine, and opioids. In this model, substance and cannabis use status were used as fixed effects. Next, we explored if the beliefs about the addictiveness of each substance differed based on the SUD severity for that given substance. For example, we tested for differences in the beliefs of the addictiveness of alcohol among the different AUD severity groups. We again employed a second mixed effects model to examine associations among SUD severity for given substances (CUD, AUD, TUD, CoCUD) and the addictiveness of that substance (cannabis, alcohol, cigarettes, cocaine, respectively). SUD severity and substance type were fixed effects. All mixed-effects models included a random intercept to account for repeated measures within participants. Type III Wald F-tests with Kenward-Roger degrees of freedom correction (Kenward and Roger 1997) were used for fixed effects. When the omnibus mixed-effects models were significant, post-hoc pairwise comparisons were conducted using estimated marginal means via the emmeans package (Lenth 2025) and the *p*-values for these post-hoc comparisons were adjusted for multiple testing corrections using the Benjamini and Hochberg method. (Benjamini and Hochberg 1995) Results are reported using estimates, 95% confidence intervals (CI), and adjusted *p*-values. To ease readability, we include only significant and relevant non-significant findings, and all statistics are reported in Supplemental Tables. An ⍺ ≤ 0.05 was considered statistically significant. All analyses were conducted using R version 4.5.1. (R Core Team 2025)

## Results

### Participant demographics and characteristics

Participants included *n* = 507 who fully completed the survey out of a total of 578 who initiated the survey. No other exclusions were made. On average, participants were 47.2 (SD = 12.2) years old, 67% female, 81% White, and 94% non-Hispanic or Latino; 62% had no college degree, and 49% reported household incomes less than $35,000. Table 1 presents demographic characteristics for Current-, Former-, and Never-CU participants. Overall, 87.2% of participants reported using cannabis currently or formerly. Across cannabis use categories, we found no significant differences in sex, race, ethnicity, and income, but observed significant differences in age, educational level, and the number of lifetime and current SUDs. Never-CU was the oldest group and was the least likely to have attended college. Current-CU reported significantly more lifetime and current SUDs. Participants reported a range of SUD severity in the past three months across substances (see Table S1). Note that the cell counts for Mild and Moderate CoUD are small relative to CUD, AUD, and TUD.

**Table 1:** Participant demographics

		Cannabis Use Status		
	Total	Current-CU	Former-CU	Never-CU
	507	182 (35.9%)	260 (51.3%)	65 (12.8%)
Age (years; Mean, SD)*	47.2 (12.2)	44.8 (10.8)^a,b^	47.9 (12.5)^a^	51.1 (13.6)^b^
Gender-Female	341 (67.3%)	114 (62.6%)	177 (68.1%)	50 (76.9%)
Race				
American Indian or Alaskan Native	10 (2.0%)	4 (2.2%)	4 (1.5%)	2 (3.1%)
Asian	8 (1.6%)	2 (1.1%)	4 (1.5%)	2 (3.1%)
Black or African American	58 (11.4%)	22 (12.1%)	21 (8.1%)	15 (23.1%)
Native Hawaiian/Pacific Islander	2 (0.4%)	1 (0.5%)	1 (0.4%)	0 (0%)
White	411 (81.1%)	147 (80.8%)	219 (84.2%)	45 (69.2%)
Multiracial	13 (2.6%)	3 (1.6%)	9 (3.5%)	1 (1.5%)
Other	5 (1.0%)	3 (1.6%)	2 (0.8%)	0 (0%)
Ethnicity—Not Hispanic or Latino	475 (93.7%)	169 (92.9%)	245 (94.2%)	61 (93.8%)
Education				
Less than High School	58 (11.4%)	21 (11.5%)	26 (10%)	11 (16.9%)
High School or Equivalent	85 (16.8%)	32 (17.6%)	45 (17.3%)	8 (12.3%)
Some College*	173 (34.1%)	64 (35.2%)^a^	98 (37.7%)^b^	11 (16.9%)^a,b^
Bachelor's Degree	98 (19.3%)	36 (19.8%)	42 (16.2%)	20 (30.8%)
Graduate or Professional Degree	93 (18.3%)	29 (15.9%)	49 (18.8%)	15 (23.1%)
Combined Household Income				
Less than $20,000	164 (32.3%)	68 (37.4%)	74 (28.5%)	22 (33.8%)
$20,000 to $34,999	86 (17.0%)	32 (17.6%)	47 (18.1%)	7 (10.8%)
$35,000 to $69,999	124 (24.5%)	41 (22.5%)	71 (27.3%)	12 (18.5%)
More than $70,000	133 (26.2%)	41 (22.5%)	68 (26.2%)	24 (36.9%)
Number Lifetime SUDs (Mean, SD)***	6.0 (3.1)	6.8 (2.7)^a^	6.3 (3.1)^b^	2.7 (1.9)^a,b^
Number Current SUDs (Mean, SD)***	2.0 (2.2)	2.8 (2.4)^a,b^	1.5 (2.0)^a^	1.5 (1.6)^b^

^a,b^Same letter superscripts within a row signify significant pairwise difference

*p* < *.*05 (*)*, p* < *.*01 (**)*, p* < *.*001 (***)

Most participants (84%) reported that medical and/or recreational use was legal where they live. Current-, Former-, and Never-CU did not differ in their opinions about whether cannabis use should be illegal, decriminalized, or recreationally legal. Current- and Former-CU, on average, initiated cannabis use during mid-teens (15.6 (4.1 SD) years). The age of initiation and the frequency of use did not differ between Current- and Former-CU. Current-CU were the most likely to have frequent (weekly to daily) encounters with people using cannabis, and were the most likely to report that at least half of their close friends and/or family use cannabis. Current-CU also reported the highest level of knowledge about cannabis and cannabis products, and were more likely to report therapeutic use than Former-CU. Former-CU were more likely to report recreational cannabis use than Current-CU (see Table 2). The most frequently reported reasons for using cannabis for therapeutic reasons were mental health conditions (48.3%; e.g. anxiety, depression, post-traumatic stress disorder, or insomnia) and chronic pain and/or inflammation (28.7%).

**Table 2:** Participant cannabis use characteristics

		Cannabis Use Status		
	Total	Current-CU	Former-CU	Never-CU
	507	182 (35.9%)	260 (51.3%)	65 (12.8%)
Age of First Cannabis Use (Mean, SD)	15.6 (4.1)	15.6 (4.1)	15.5 (4.1)	-
Cannabis Use Frequency				
A few times per month or less	174 (39.4%)	69 (37.9%)	105 (40.4%)	-
Weekly to daily	268 (60.6%)	113 (62.1%)	155 (59.6%)	-
Cannabis Use Purpose				
Recreational***	233 (46.0%)	45 (24.7%)^a^	188 (72.3%)^a^	-
Medical—With a Prescription**	64 (12.6%)	45 (24.7%)^a^	19 (7.3%)^a^	-
Medical—No Prescription***	145 (28.6%)	92 (50.5%)^a^	53 (20.4%)^a^	-
Cannabis Knowledge (Mean, SD)	1.7 (1.2)	2.3 (1.1)^a,b^	1.6 (1.2)^a,c^	0.9 (1.0)^b,c^
How many close friends/family use cannabis?				
At least half**	210 (41.4%)	111 (61.0%)^a,b^	86 (33.1%)^a^	13 (20.0%)^b^
A few or none**	279 (55.0%)	65 (35.7%)^a,b^	166 (63.8%)^a^	48 (73.8%)^b^
Unsure	18 (3.6%)	6 (3.3%)	8 (3.1%)	4 (6.2%)
How often are you around people using cannabis?				
A few times per month or less**	329 (64.9%)	80 (44.0%)^a,b^	193 (74.2%)^a^	56 (86.2%)^b^
Weekly to daily**	178 (35.1%)	102 (56.0%)^a,b^	67 (25.8%)^a^	9 (13.8%)^b^
Cannabis Legal Status at Home				
Illegal or decriminalized	82 (16.2%)	32 (17.6%)	43 (16.5%)	7 (10.8%)
Legal for medical use	119 (23.5%)	40 (22.0%)	65 (25.0%)	14 (21.5%)
Legal for medical and recreational use	306 (60.4%)	110 (60.4%)	152 (58.5%)	44 (67.7%)
Beliefs about legality				
Illegal or decriminalized	117 (23.1%)	46 (25.3%)	57 (21.9%)	14 (21.5%)
Legal for medical use only**	42 (8.3%)	2 (1.1%)^a,b^	27 (10.4%)^a^	13 (20.0%)^b^
Regulated like alcohol and tobacco	200 (39.4%)	85 (46.7%)	94 (36.2%)	21 (32.3%)
Unregulated like non-prescription herbs**	101 (19.9%)	47 (25.8%)^a^	51 (19.6%)^b^	3 (4.6%)^a,b^
No Opinion**	47 (9.3%)	2 (1.1%)^a,b^	31 (11.9%)^a^	14 (21.5%)^b^

^a,b,c^Same letter superscripts within a row signify significant pairwise differences

*p* < *.05* (*)*, p* < *.01* (**)*, p* < *.001* (***)

### Beliefs about cannabis

**Fig. 1:**
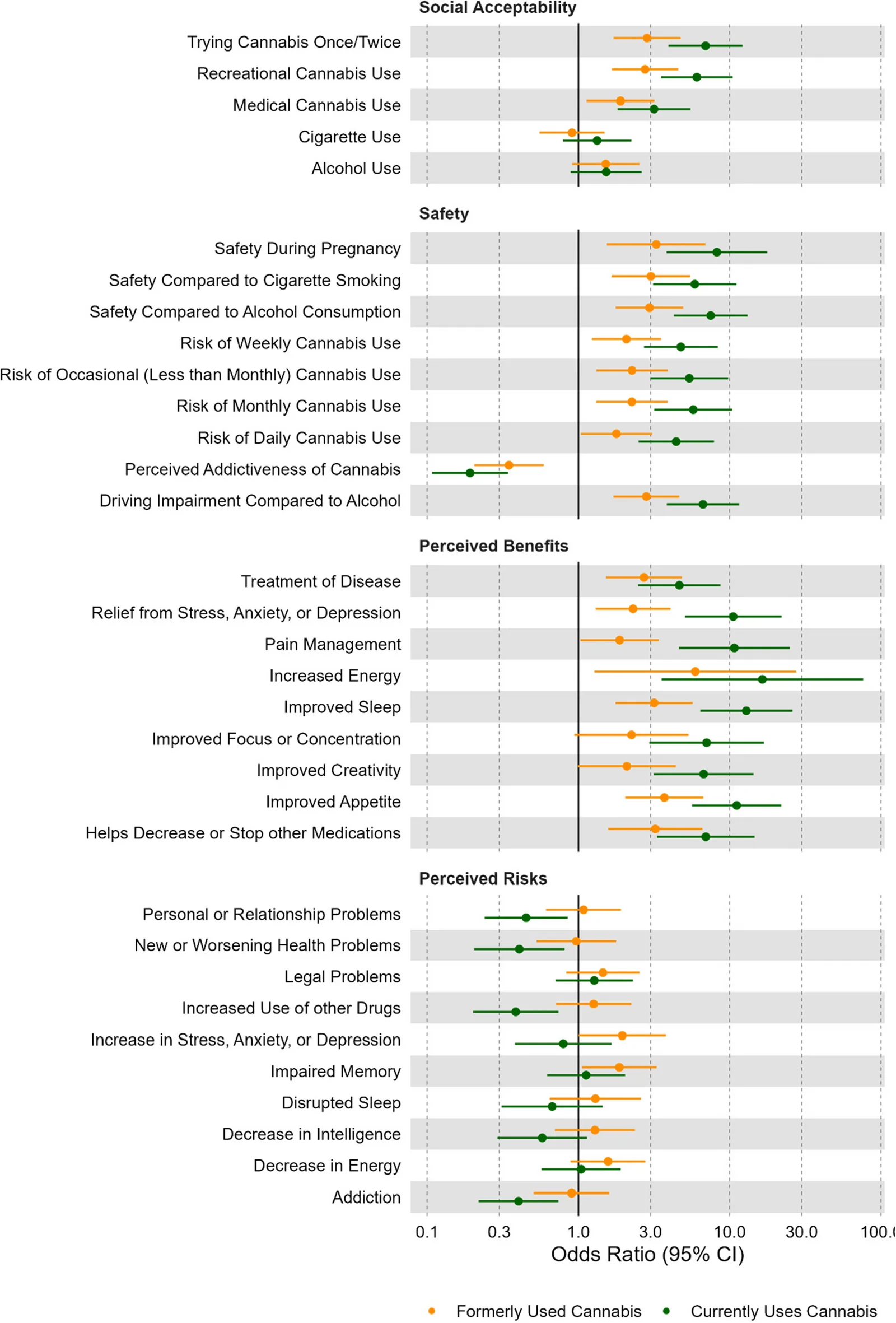
Beliefs about cannabis use. Forest plot showing results of logistic regression models examining the relations between cannabis use status and four belief categories. Odds ratios and 95% confidence intervals are plotted for each outcome, stratified by cannabis use status. The reference category at Odds Ratio = 1 is “Never Uses Cannabis”. Table S2 includes all Odds ratios, standard errors, 95% confidence intervals, and adjusted *p*-values for each pairwise comparison

Relations between cannabis use status and beliefs about cannabis are reported in Fig. 1. Odds ratios, standard error estimates, 95% confidence intervals, and adjusted *p*-values for each pairwise comparison are included in Table S2.

#### Social acceptability

In general, Current- and Former-CU were more likely to believe cannabis is socially acceptable than Never-CU. Compared to Never-CU, Current- and Former-CU were more likely to believe that recreational cannabis use, trying cannabis once or twice for non-therapeutic reasons, and therapeutic use are socially acceptable. In contrast, no significant differences in the social acceptability of alcohol and cigarette use by cannabis use status were observed.

#### Safety

Current- and Former-CU were more likely to believe that cannabis use is safe than Never-CU. Compared to Never-CU, Current- and Former-CU were more likely to believe cannabis use was somewhat or completely safe during pregnancy; somewhat or much safer than alcohol use while driving; that smoking one cannabis joint per day is somewhat or much safer than drinking one glass of wine per day, and that smoking one cannabis joint is somewhat more safe or much safer than smoking a few cigarettes per day.

#### Benefits

Current- and Former-CU were more likely to believe that cannabis use is beneficial than Never-CU. Current-CU were more likely than Former- and Never-CU to believe that cannabis use provides all of the nine listed benefits. Current-CU were significantly more likely to believe cannabis use improved focus and concentration than both Former- and Never-CU, but Former-CU were no more likely to endorse this belief than Never-CU.

#### Risks

Beliefs about the risks associated with cannabis use by cannabis use status did not follow the same pattern as beliefs about acceptability, safety, and benefits. Compared to Former-CU, Current-CU were less likely to believe that cannabis use was linked with nearly all the 10 listed risks with exceptions of legal problems and decreased energy. Compared to Never-CU, Current-CU were less likely to believe cannabis use is linked with addiction, increased use of other drugs, personal or relationship problems, and new or worsening health problems, but did not differ from Never-CU in their beliefs about impaired memory; decrease in intelligence; decrease in energy; increase in stress, anxiety or depression; or disrupted sleep. Compared to Never-CU, Former-CU were more likely to believe that cannabis use is linked with impaired memory, but did not differ in their beliefs about cannabis being linked with most risks including addiction, increased use of other drugs, personal or relationship problems, decrease in intelligence, new or worsening health problems, disrupted sleep, and increase in stress, anxiety, or depression. Current-, Former-, and Never-CU did not differ in their belief that cannabis is linked with legal problems (see Table S2).

#### Addictiveness

**Fig. 2:**
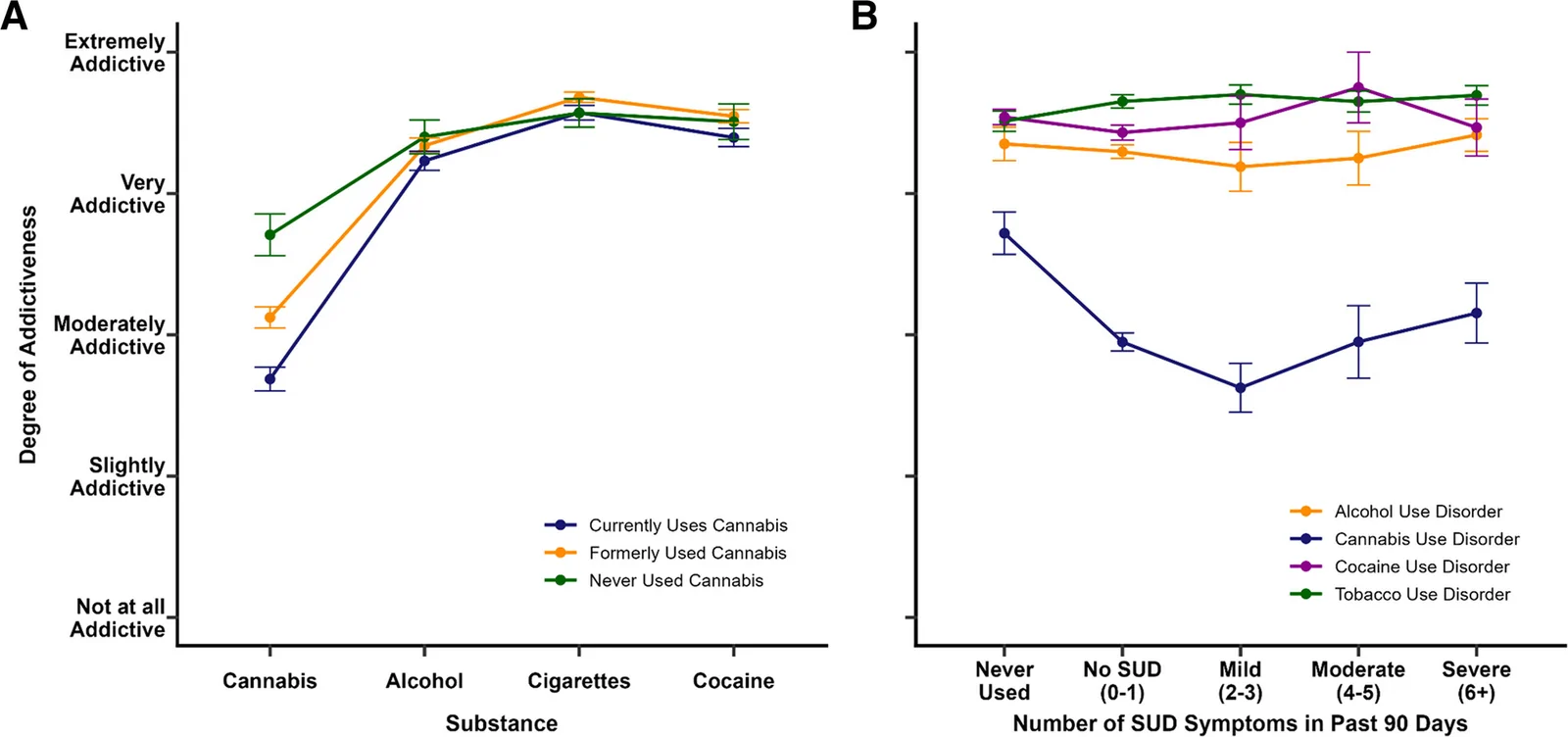
A comparison of beliefs about the degree of addictiveness of cannabis, alcohol, cigarettes, and cocaine. Line plot indicating the (**A**) mean addictiveness of cannabis, alcohol, cigarettes, and cocaine stratified by current cannabis use, former cannabis use, and never cannabis use. **B** Mean addictiveness of cannabis, alcohol, tobacco, and cocaine. The groups are stratified by DSM-5 3-month severity category (number of symptoms) for alcohol, cannabis, cocaine, and tobacco. That is, the orange line represents individuals grouped by their severity of alcohol use disorder; the blue line represents individuals grouped by their severity of cannabis use disorder; the purple line represents individuals grouped by their severity of cocaine use disorder; and the green line represents individuals grouped by their severity of tobacco use disorder. Note that the cell counts for Mild and Moderate CoUD are small relative to CUD, AUD, and TUD. Error bars represent standard errors of the mean. Tables S3 and S4 show regression estimates, standard errors, 95% confidence intervals, and adjusted *p*-values for each pairwise comparison

All three cannabis use groups differed in their beliefs about the degree to which cannabis is addictive (Fig. 2A). Current-CU believed cannabis was slightly to moderately addictive while Former- and Never-CU believed cannabis was moderately to very addictive. All cannabis use groups believed cannabis was less addictive than alcohol, cigarettes, and cocaine. In contrast, no significant differences were found among the three groups in beliefs about the addictiveness of alcohol, cigarettes, and cocaine (Table S3). Current-CU also believed alcohol was less addictive than cigarettes, and cocaine was less addictive than cigarettes. Former-CU believed alcohol was less addictive than cigarettes and cocaine. Among Never-CU, no significant differences were found in beliefs about the addictiveness of alcohol, cigarettes, and cocaine. Among all three groups, no significant differences were found in beliefs about the addictiveness of cigarettes.

Among participants with CUD (*n* = 91; see Table S1), beliefs about the degree of addictiveness of cannabis were related to the severity of CUD, while beliefs about the addictiveness of other substances were independent of SUD severity. Beliefs about the degree of addictiveness of cannabis were significantly lower than all other substances for each severity level. Participants who never used cannabis (analogous to Never-CU; Fig. 2B) believed cannabis was more addictive than participants who used cannabis, regardless of CUD severity (Table S4). Interestingly, participants with mild CUD believed cannabis was the least addictive. Among those in the no SUD group, beliefs about the addictiveness of alcohol were significantly lower than that of cigarettes. In contrast, significant differences in the beliefs about the addictiveness of alcohol were not observed by AUD severity; significant differences in the beliefs about the addictiveness of cocaine were not observed by CoUD severity; and significant differences in beliefs about the addictiveness of cigarettes were not observed by TUD severity.

## Discussion

This study aimed to describe cannabis use among a large sample of people in recovery from common SUDS, explored beliefs about social acceptability, safety, benefits, risks, and addictiveness of cannabis among participants who currently, formerly, or never used cannabis, and finally, compared beliefs about the addictiveness of cannabis with that of other substances. We hypothesized that most participants would have experience using cannabis, and that participants who currently use cannabis would have more positive beliefs about cannabis use than those who formerly or never used cannabis. Our findings support our aims and expand upon the broader literature to advance our understanding of cannabis use among people in recovery.

Cannabis use is highly prevalent, and among people in recovery, its use has been proposed as a therapeutic intervention for treating anxiety, depression, and other mental health disorders as well as a substitute for other SUDS. (Leung et al. 2022) As expected, most participants (87.2%) had experience with cannabis, similar to that reported in other studies. (Jones et al. 2020) Further, our findings echo the therapeutic utilization of cannabis in the community, as evidenced by the three-quarters (75%) of the sample that reported using cannabis for therapeutic purposes, including the management of other SUDs. Despite this high prevalence of therapeutic use, there is insufficient evidence for the efficacy and safety of cannabis for the treatment of substance use disorders, and other mental health presentations (e.g., anxiety, post-traumatic stress disorder, ADHD, bipolar disorder, and obsessive–compulsive disorder) among people with SUDs. (Wilson et al. 2026) In the face of lacking evidentiary support for the efficacy and safety of therapeutic cannabis, reasons why individuals in recovery continue to use cannabis remain unanswered, and could be explained by beliefs about cannabis.

Participants with cannabis experience, either currently or formerly, held the most favorable beliefs about cannabis overall. While beliefs about cannabis use have been documented in community samples, (Doggett et al. 2025; Mennis et al. 2023) this is the first report of cannabis beliefs for individuals in SUD recovery. Individuals in SUD recovery have more favorable beliefs regarding social acceptability and safety, in contrast to other studies. (Doggett et al. 2025) Moreover, these positive beliefs also extended to use during pregnancy and driving under the influence, which is concerning.e.g., (Young-Wolff et al. 2024; Pearlson et al. 2021) Former-CU and Never-CU believed cannabis use causes impaired memory; decreases in intelligence; decreases in energy; increases in stress, anxiety or depression; and disrupted sleep. Perhaps from experience, Former-CU were the most likely to believe cannabis use causes impaired memory and increase in stress, anxiety, or depression.

Although cannabis use status was associated with beliefs about the addictiveness of cannabis, all participants were generally in agreement that cannabis is less addictive than alcohol, cigarettes, and cocaine. In addition, among participants with CUD, beliefs about the addictiveness of cannabis were different than for any other substance. Even those with severe CUD believed cannabis was less addictive than other substances. Current-CU reported nearly twice the number of current SUDs than Never-CU, which supports prior research among people in recovery suggesting that cannabis use is linked with addiction to other substances. (Subbaraman et al. 2025; Warnock et al. 2024) Importantly, these differences in addictiveness also are not apparently due to unfamiliarity with cannabis use. The reasons why all participants view cannabis as less addictive than other substances is unclear, but is consistent with the general public. (American Psychiatric Association 2023). These beliefs might be influenced by legalization, unclear boundaries between therapeutic and recreational use, increased exposure to and availability of cannabis products, sociocultural beliefs, marketing and framing of cannabis use, or long-standing stereotypes about cannabis use as harmless fun. (Reid, M.^ 2020^). Or, perhaps self-selection occurs where people whose current cannabis use has not caused problems in their lives and has led to better functioning and greater well-being (Eddie et al. 2022) continue to use cannabis, whereas people for which cannabis use has severely impacted their life have discontinued its use.

We speculate that participant’s beliefs about the addictiveness of cannabis might be accurate. Although cannabis use conforms to the same neuro-behavioral models of addiction as other substances, it does so less robustly. (Peters et al. 2021; Zehra et al. 2018) CUD has all of the hallmarks of other SUDs (Budney et al. 2019), but generally manifests in milder forms than other SUDs. (Budney 2006; Peer et al. 2013; Shmulewitz et al. 2015) Despite this, even though estimation and sampling methods vary significantly from study to study, the rank order of the most prevalent SUDs (TUD notwithstanding) is consistently found to be AUD first, followed by CUD, with opioid use disorder, and stimulant use disorders (i.e., cocaine, methamphetamine, etc.) following behind. (Mojtabai 2022; Ringeisen et al. 2025; Volkow et al. 2021) Thus, despite CUD having an overall greater prevalence, opioids and cocaine are still seen as more harmful and dangerous. Unlike opioids and cocaine, cannabis-related deaths are rare. (Rock et al. 2022) Further, the clinical management of cannabis withdrawal and CUD includes no medications, and is limited to psychoeducation and counseling which might give the impression that CUD is less addictive. (Connor et al. 2022) Cannabis withdrawal symptoms might be unrecognized or dismissed, given that there are no effective treatment strategies. In this study, perhaps the positive correlation between CUD severity and addictiveness simply reflects participants' experiences. Further studies investigating the addictiveness of cannabis among SUD type could elucidate knowledge regarding personal experiences.

In contrast, socially-mediated perceptions may facilitate the under-estimation of the risks of using cannabis. Sociological research has suggested that cannabis use has undergone a process of “normalization,” in which behaviors once seen as immoral or deviant become more integrated into mainstream social contexts. (Duff et al. 2012; Hathaway et al. 2011; Parker and Aldrige 1998; Parker 2005) Likely as a result, representations of cannabis use in films, media, and popular culture have evolved and now more regularly depict it as a relatively ordinary, and sometimes humorous, leisure activity. Conversely, other drugs like cocaine, methamphetamine, or heroin are more often associated with addiction, criminality, violence, and dehumanizing language. (Giorgi et al. 2023; Rawstorne et al. 2020) In addition, although cannabis may have some medicinal utility when applied to specific ailments, exposure to cannabis marketing has been associated with increases in cannabis use, more positive attitudes about cannabis, lower harm perceptions, and more interest in obtaining a medical certification for use. (Cohn et al. 2023) These factors likely work in combination to alter public perceptions which may ultimately result in the trivialization of addiction-related risks and harmful outcomes of cannabis use such as impaired cognitive functions, increased risk of psychiatric disorders, and motor vehicle accidents. (Hall and Degenhardt 2009; Hall et al. 2020).

We also speculate that perhaps beliefs about the addictiveness of cannabis might change if people in recovery gain more experience with more potent cannabis products. Higher cannabis product potency is related to the development of CUD, other health concerns, and perceived addictiveness. (Arterberry et al. 2019) In the past two decades, the potency of Delta-9-tetrahydrocannabinol (THC) concentrations in illegal cannabis increased from 3.96% to 16.14%, (2024) and recent cannabis products in dispensaries can have THC concentrations of more than 40%. (Cash et al. 2020) Future studies should explore the role of cannabis potency as harm profiles may vary substantially according to product strength and bioavailability.

The strengths of this study include a large, community-based convenience sample of people in recovery, many of whom represent real-world polysubstance use; however, study conclusions should be interpreted in light of several limitations. First, self-report research has the potential to engender social desirability, memory and/or recall bias, misinterpretation of questions, and tendencies to exaggerate or downplay symptoms. For example, here we used a self-report checklist of DSM-5 symptoms to assess SUD severity which may have inflated the number of participants meeting diagnostic criteria for SUD(s). In addition, these findings are also cross-sectional and are not from a nationally representative sample so are not generalizable to all people who have or have had SUDs, or consider themselves in recovery. Finally, beliefs about cannabis use among people in recovery might change over time, especially if more personalized and non-abstinence approaches to treatment and recovery are more widely accepted. Future research might examine beliefs and cannabis use in a nationally representative sample and include longitudinal assessments that evaluate changes in cannabis use and beliefs.

## Conclusions

In summary, our findings underscore the importance of recognizing heterogeneity in cannabis use among people in recovery. In addition, they seem to signify that cannabis, relative to other substances, occupies a unique perceptual position among people in recovery. People in recovery might correctly perceive cannabis to be less addictive than other substances. However, these beliefs might change if they become more knowledgeable about risks and safety, they experience products with higher potencies, or new clinical guidelines are developed for treating CUD. As cannabis regulation evolves, understanding how these perceptions may influence substance use decisions and recovery trajectories will be increasingly important. Greater attention to how cannabis is perceived within recovery contexts and treatment settings may help clarify if or when cannabis use may function as a safer alternative to other substance use as opposed to when it might pose a risk to sustained recovery.

## Supplementary Information

Supplementary Material 1.

## Data Availability

The datasets used and/or analysed during the current study are available from the corresponding author on reasonable request.
