# Supplementary material for: Cannabis use and beliefs among people in recovery from substance use disorders
Source: J Cannabis Res. 2026 Jul 3;8:102. doi: 10.1186/s42238-026-00461-5 (PMC13632430; doi:10.1186/s42238-026-00461-5)
Supplement: Supplementary file 1 [file 42238_2026_461_MOESM1_ESM.docx]

**Supplemental Tables**

| **Table S1**. Proportion of DSM-5 substance use disorder diagnostic criteria symptoms endorsed per substance in the past three months | | | | | | | | | | | |
| --- | --- | --- | --- | --- | --- | --- | --- | --- | --- | --- | --- |
|  | Using more1 | Attempts to control use2 | Time spent using3 | Craving4 | Neglecting major roles5 | Interpersonal problems6 | Giving up activities7 | Hazardous use8 | Problems related to use9 | Tolerance10 | Withdrawal11 |
| Cannabis (n=442) | 14.5% | 11.5% | 9.5% | 23.1% | 7.9% | 7.0% | 7.5% | 8.1% | 10.6% | 14.5% | 7.2% |
| Alcohol (n=430) | 19.5% | 19.8% | 16.7% | 31.6% | 11.9% | 13.5% | 13.0% | 8.8% | 16.0% | 13.7% | 13.0% |
| Nicotine /  Tobacco (n=374) | 38.8% | 42.0% | 19.8% | 62.6% | 11.8% | 18.2% | 9.4% | 13.9% | 34.8% | 29.7% | 30.5% |
| Cocaine (n=274) | 12.0% | 11.3% | 9.9% | 15.3% | 9.9% | 11.3% | 9.5% | 7.3% | 10.9% | 11.3% | 9.9% |
| Opioids (n=281) | 10.7% | 14.9% | 10.7% | 23.5% | 10.3% | 9.6% | 9.6% | 7.5% | 10.7% | 11.4% | 12.5% |
| ^1^When you used each of the following substances, did you end up using more OR for a longer amount of time than you planned?  ^2^Did you repeatedly want to reduce or control your use OR did you try to cut down or control your use of the following substance(s), but failed?  ^3^On the days that you used each of the following substance(s), did you spend substantial time either obtaining, using, or recovering from its effects?  ^4^Did you ever experience cravings, or a strong desire or urge to use the following substance(s)?  ^5^Did you spend less time meeting your responsibilities at work, at school, or at home, because of your repeated use of the following substances?  ^6^If your use of each of the following substance(s) caused problems with your family or other people, did you still keep using it?  ^7^Did you reduce OR give up social, or recreational activities/hobbies because of your use of each of the following substance(s)?  ^8^Did you use the following substance(s) more than once in any situation where you or others were physically at risk (for example, driving a car, riding a motorcycle, using machinery, boating, etc.)?  ^9^Did you continue to use the following substance(s) even though it was clear that it caused physical or mental problems or made these problems worse?  ^10^Did you ever need to use more of the following substances to get the same effect that you did when you first started using it? AND/OR did you ever get much less of an effect when you continued to use the same amount?  ^11^When you reduced or stopped using the following substances, did you have withdrawal symptoms (aches, shaking, fever, weakness, diarrhea, nausea, sweating,heart pounding, difficulty sleeping, or feeling agitated, anxious, irritable, or depressed)? (AND/OR) Did you continue to use it to keep yourself from getting sick (withdrawal symptoms) or so that you would feel better? | | | | | | | | | | | |

| **Table S2**. Post-hoc pairwise comparisons from logistic regression models | | | | | |
| --- | --- | --- | --- | --- | --- |
|  | | | | | |
| Domain/Outcome | Odds Ratio | SE | 95% CI Lower | 95% CI Upper | *p* value |
| **Social Acceptability** | | | | | |
| ***Recreational Cannabis Use*** |  |  |  |  |  |
| Current vs. Former | 2.2 | 0.18 | 1.54 | 3.14 | < .001 |
| Current vs. Never | 6.07 | 0.28 | 3.52 | 10.47 | < .001 |
| Former vs. Never | 2.76 | 0.26 | 1.66 | 4.58 | < .001 |
| ***Trying Cannabis Once/Twice*** |  |  |  |  |  |
| Current vs. Former | 2.44 | 0.19 | 1.66 | 3.57 | < .001 |
| Current vs. Never | 6.93 | 0.29 | 3.95 | 12.18 | < .001 |
| Former vs. Never | 2.85 | 0.26 | 1.71 | 4.73 | < .001 |
| ***Medical Cannabis Use*** |  |  |  |  |  |
| Current vs. Former | 1.67 | 0.19 | 1.15 | 2.41 | 0.010 |
| Current vs. Never | 3.16 | 0.28 | 1.82 | 5.51 | < .001 |
| Former vs. Never | 1.9 | 0.26 | 1.13 | 3.18 | 0.015 |
| ***Alcohol Use*** |  |  |  |  |  |
| Current vs. Former | 1.01 | 0.17 | 0.71 | 1.42 | 0.969 |
| Current vs. Never | 1.53 | 0.28 | 0.89 | 2.62 | 0.185 |
| Former vs. Never | 1.52 | 0.26 | 0.91 | 2.53 | 0.185 |
| ***Cigarette Use*** |  |  |  |  |  |
| Current vs. Former | 1.46 | 0.18 | 1.04 | 2.07 | 0.088 |
| Current vs. Never | 1.33 | 0.27 | 0.79 | 2.24 | 0.426 |
| Former vs. Never | 0.91 | 0.25 | 0.55 | 1.49 | 0.701 |
| **Safety** | | | | | |
| ***Risk of Occasional (Less than Monthly) Cannabis Use*** |  |  |  |  |  |
| Current vs. Former | 2.39 | 0.19 | 1.64 | 3.5 | < .001 |
| Current vs. Never | 5.41 | 0.3 | 3 | 9.78 | < .001 |
| Former vs. Never | 2.26 | 0.28 | 1.31 | 3.9 | < .001 |
| ***Risk of Monthly Cannabis Use*** |  |  |  |  |  |
| Current vs. Former | 2.55 | 0.19 | 1.74 | 3.73 | < .001 |
| Current vs. Never | 5.75 | 0.3 | 3.18 | 10.39 | < .001 |
| Former vs. Never | 2.26 | 0.28 | 1.31 | 3.89 | < .001 |
| ***Risk of Weekly Cannabis Use*** |  |  |  |  |  |
| Current vs. Former | 2.29 | 0.18 | 1.6 | 3.28 | < .001 |
| Current vs. Never | 4.76 | 0.29 | 2.71 | 8.35 | < .001 |
| Former vs. Never | 2.08 | 0.27 | 1.23 | 3.51 | 0.006 |
| ***Risk of Daily Cannabis Use*** |  |  |  |  |  |
| Current vs. Former | 2.48 | 0.18 | 1.75 | 3.53 | < .001 |
| Current vs. Never | 4.43 | 0.29 | 2.5 | 7.87 | < .001 |
| Former vs. Never | 1.78 | 0.28 | 1.04 | 3.07 | 0.036 |
| ***Perceived Addictiveness of Cannabis*** |  |  |  |  |  |
| Current vs. Former | 0.55 | 0.2 | 0.38 | 0.81 | < .001 |
| Current vs. Never | 0.19 | 0.29 | 0.11 | 0.34 | < .001 |
| Former vs. Never | 0.35 | 0.27 | 0.21 | 0.59 | < .001 |
| ***Safety During Pregnancy*** |  |  |  |  |  |
| Current vs. Former | 2.52 | 0.19 | 1.74 | 3.64 | < .001 |
| Current vs. Never | 8.24 | 0.39 | 3.86 | 17.59 | < .001 |
| Former vs. Never | 3.27 | 0.38 | 1.55 | 6.89 | < .001 |
| ***Driving Impairment Compared to Alcohol*** |  |  |  |  |  |
| Current vs. Former | 2.37 | 0.19 | 1.65 | 3.41 | < .001 |
| Current vs. Never | 6.67 | 0.28 | 3.85 | 11.55 | < .001 |
| Former vs. Never | 2.81 | 0.26 | 1.71 | 4.64 | < .001 |
| ***Safety Compared to Alcohol Consumption*** |  |  |  |  |  |
| Current vs. Former | 2.54 | 0.18 | 1.78 | 3.63 | < .001 |
| Current vs. Never | 7.5 | 0.29 | 4.28 | 13.13 | < .001 |
| Former vs. Never | 2.95 | 0.26 | 1.77 | 4.93 | < .001 |
| ***Safety Compared to Cigarette Smoking*** |  |  |  |  |  |
| Current vs. Former | 1.95 | 0.2 | 1.31 | 2.9 | < .001 |
| Current vs. Never | 5.88 | 0.32 | 3.13 | 11.05 | < .001 |
| Former vs. Never | 3.01 | 0.3 | 1.66 | 5.46 | < .001 |
| **Perceived Benefits** |  |  |  |  |  |
| ***Pain Management*** |  |  |  |  |  |
| Current vs. Former | 5.73 | 0.36 | 2.82 | 11.65 | < .001 |
| Current vs. Never | 10.74 | 0.43 | 4.64 | 24.84 | < .001 |
| Former vs. Never | 1.87 | 0.3 | 1.03 | 3.4 | 0.039 |
| ***Treatment of Disease*** |  |  |  |  |  |
| Current vs. Former | 1.71 | 0.21 | 1.12 | 2.61 | 0.012 |
| Current vs. Never | 4.65 | 0.32 | 2.49 | 8.68 | < .001 |
| Former vs. Never | 2.71 | 0.29 | 1.52 | 4.83 | 0.001 |
| ***Relief From Stress, Anxiety, or Depression*** |  |  |  |  |  |
| Current vs. Former | 4.59 | 0.3 | 2.55 | 8.27 | < .001 |
| Current vs. Never | 10.56 | 0.37 | 5.08 | 21.97 | < .001 |
| Former vs. Never | 2.3 | 0.29 | 1.3 | 4.06 | < .001 |
| ***Improved Appetite*** |  |  |  |  |  |
| Current vs. Former | 3.01 | 0.24 | 1.86 | 4.86 | < .001 |
| Current vs. Never | 11.14 | 0.35 | 5.66 | 21.93 | < .001 |
| Former vs. Never | 3.7 | 0.3 | 2.05 | 6.69 | < .001 |
| ***Improved Sleep*** |  |  |  |  |  |
| Current vs. Former | 4.07 | 0.26 | 2.42 | 6.84 | < .001 |
| Current vs. Never | 12.9 | 0.36 | 6.42 | 25.93 | < .001 |
| Former vs. Never | 3.17 | 0.3 | 1.77 | 5.68 | < .001 |
| ***Helps Decrease or Stop other Medications*** |  |  |  |  |  |
| Current vs. Former | 2.16 | 0.21 | 1.44 | 3.22 | < .001 |
| Current vs. Never | 6.96 | 0.38 | 3.33 | 14.52 | < .001 |
| Former vs. Never | 3.23 | 0.36 | 1.59 | 6.56 | < .001 |
| ***Improved Creativity*** |  |  |  |  |  |
| Current vs. Former | 3.22 | 0.21 | 2.14 | 4.84 | < .001 |
| Current vs. Never | 6.73 | 0.38 | 3.17 | 14.29 | < .001 |
| Former vs. Never | 2.09 | 0.38 | 1 | 4.38 | 0.050 |
| ***Improved Focus or Concentration*** |  |  |  |  |  |
| Current vs. Former | 3.14 | 0.22 | 2.05 | 4.8 | < .001 |
| Current vs. Never | 7.04 | 0.44 | 2.98 | 16.66 | < .001 |
| Former vs. Never | 2.24 | 0.44 | 0.95 | 5.29 | 0.065 |
| ***Increased Energy*** |  |  |  |  |  |
| Current vs. Former | 2.78 | 0.24 | 1.75 | 4.41 | < .001 |
| Current vs. Never | 16.46 | 0.74 | 3.83 | 70.7 | < .001 |
| Former vs. Never | 5.93 | 0.74 | 1.38 | 25.52 | 0.017 |
| **Perceived Risks** | | | | | |
| ***Legal Problems*** |  |  |  |  |  |
| Current vs. Former | 0.88 | 0.2 | 0.59 | 1.29 | 0.508 |
| Current vs. Never | 1.27 | 0.3 | 0.71 | 2.29 | 0.508 |
| Former vs. Never | 1.45 | 0.28 | 0.83 | 2.53 | 0.508 |
| ***Addiction*** |  |  |  |  |  |
| Current vs. Former | 0.45 | 0.2 | 0.3 | 0.66 | < .001 |
| Current vs. Never | 0.4 | 0.31 | 0.22 | 0.74 | 0.005 |
| Former vs. Never | 0.9 | 0.29 | 0.51 | 1.6 | 0.723 |
| ***Impaired Memory*** |  |  |  |  |  |
| Current vs. Former | 0.6 | 0.2 | 0.41 | 0.89 | 0.036 |
| Current vs. Never | 1.12 | 0.3 | 0.62 | 2.03 | 0.697 |
| Former vs. Never | 1.86 | 0.29 | 1.06 | 3.28 | 0.047 |
| ***Increased Use of other Drugs*** |  |  |  |  |  |
| Current vs. Former | 0.31 | 0.24 | 0.19 | 0.48 | < .001 |
| Current vs. Never | 0.39 | 0.33 | 0.2 | 0.74 | 0.006 |
| Former vs. Never | 1.26 | 0.29 | 0.71 | 2.23 | 0.426 |
| ***Personal or Relationship Problems*** |  |  |  |  |  |
| Current vs. Former | 0.42 | 0.22 | 0.27 | 0.65 | < .001 |
| Current vs. Never | 0.45 | 0.32 | 0.24 | 0.85 | 0.020 |
| Former vs. Never | 1.08 | 0.29 | 0.61 | 1.91 | 0.787 |
| ***Decrease in Intelligence*** |  |  |  |  |  |
| Current vs. Former | 0.45 | 0.24 | 0.28 | 0.72 | 0.003 |
| Current vs. Never | 0.58 | 0.35 | 0.29 | 1.14 | 0.168 |
| Former vs. Never | 1.29 | 0.31 | 0.7 | 2.36 | 0.415 |
| ***Decrease in Energy*** |  |  |  |  |  |
| Current vs. Former | 0.66 | 0.2 | 0.45 | 0.98 | 0.122 |
| Current vs. Never | 1.04 | 0.31 | 0.57 | 1.9 | 0.893 |
| Former vs. Never | 1.57 | 0.29 | 0.89 | 2.77 | 0.178 |
| ***New or Worsening Health Problems*** |  |  |  |  |  |
| Current vs. Former | 0.42 | 0.25 | 0.26 | 0.69 | 0.002 |
| Current vs. Never | 0.41 | 0.35 | 0.2 | 0.81 | 0.015 |
| Former vs. Never | 0.97 | 0.31 | 0.53 | 1.77 | 0.917 |
| ***Increase in Stress, Anxiety, or Depression*** |  |  |  |  |  |
| Current vs. Former | 0.41 | 0.24 | 0.25 | 0.65 | 0.001 |
| Current vs. Never | 0.79 | 0.37 | 0.38 | 1.65 | 0.538 |
| Former vs. Never | 1.95 | 0.34 | 1.01 | 3.78 | 0.072 |
| ***Disrupted Sleep*** |  |  |  |  |  |
| Current vs. Former | 0.52 | 0.27 | 0.31 | 0.87 | 0.040 |
| Current vs. Never | 0.67 | 0.39 | 0.31 | 1.44 | 0.460 |
| Former vs. Never | 1.29 | 0.35 | 0.65 | 2.58 | 0.463 |

| **Table S3**. Post-hoc pairwise comparisons from perceived addictiveness multivariate mixed effects linear model | | | | | | |
| --- | --- | --- | --- | --- | --- | --- |
| Comparison | Estimate | SE | 95% CI Lower | 95% CI Upper | *t ratio* | *p value* |
| **Cannabis** | | | | | | |
| Current vs. Former | -0.44 | 0.08 | -0.73 | -0.14 | -5.20 | < .001 |
| Current vs. Never | -1.02 | 0.13 | -1.46 | -0.58 | -8.13 | < .001 |
| Former vs. Never | -0.58 | 0.12 | -1.01 | -0.16 | -4.85 | < .001 |
| **Alcohol** | | | | | | |
| Current vs. Former | -0.11 | 0.08 | -0.40 | 0.19 | -1.28 | 0.256 |
| Current vs. Never | -0.17 | 0.13 | -0.61 | 0.27 | -1.35 | 0.233 |
| Former vs. Never | -0.06 | 0.12 | -0.48 | 0.36 | -0.51 | 0.680 |
| **Cigarettes** | | | | | | |
| Current vs. Former | -0.11 | 0.08 | -0.40 | 0.18 | -1.30 | 0.250 |
| Current vs. Never | 0.00 | 0.13 | -0.44 | 0.44 | 0.02 | 0.995 |
| Former vs. Never | 0.11 | 0.12 | -0.31 | 0.53 | 0.93 | 0.423 |
| **Cocaine** | | | | | | |
| Current vs. Former | -0.15 | 0.08 | -0.44 | 0.14 | -1.79 | 0.111 |
| Current vs. Never | -0.11 | 0.13 | -0.55 | 0.33 | -0.89 | 0.434 |
| Former vs. Never | 0.04 | 0.12 | -0.38 | 0.46 | 0.32 | 0.811 |
| **Opioids** | | | | | | |
| Current vs. Former | -0.12 | 0.08 | -0.42 | 0.17 | -1.46 | 0.208 |
| Current vs. Never | 0.13 | 0.13 | -0.31 | 0.57 | 1.02 | 0.374 |
| Former vs. Never | 0.25 | 0.12 | -0.17 | 0.67 | 2.07 | 0.062 |
| **Current** | | | | | | |
| Cannabis vs. Alcohol | -1.54 | 0.07 | -1.80 | -1.28 | -20.76 | < .001 |
| Cannabis vs. Cigarettes | -1.88 | 0.07 | -2.14 | -1.62 | -25.34 | < .001 |
| Cannabis vs. Cocaine | -1.71 | 0.07 | -1.97 | -1.45 | -22.97 | < .001 |
| Cannabis vs. Opioids | -1.99 | 0.07 | -2.25 | -1.73 | -26.82 | < .001 |
| Alcohol vs. Cigarettes | -0.34 | 0.07 | -0.60 | -0.08 | -4.58 | < .001 |
| Alcohol vs. Cocaine | -0.16 | 0.07 | -0.43 | 0.10 | -2.22 | 0.045 |
| Alcohol vs. Opioids | -0.45 | 0.07 | -0.71 | -0.19 | -6.06 | < .001 |
| Cigarettes vs. Cocaine | 0.18 | 0.07 | -0.08 | 0.44 | 2.36 | 0.032 |
| Cigarettes vs. Opioids | -0.11 | 0.07 | -0.37 | 0.15 | -1.48 | 0.204 |
| Cocaine vs. Opioids | -0.29 | 0.07 | -0.55 | -0.03 | -3.84 | < .001 |
| **Former** | | | | | | |
| Cannabis vs. Alcohol | -1.22 | 0.06 | -1.43 | -1.00 | -19.53 | < .001 |
| Cannabis vs. Cigarettes | -1.56 | 0.06 | -1.78 | -1.34 | -25.03 | < .001 |
| Cannabis vs. Cocaine | -1.42 | 0.06 | -1.64 | -1.21 | -22.87 | < .001 |
| Cannabis vs. Opioids | -1.68 | 0.06 | -1.90 | -1.46 | -27.01 | < .001 |
| Alcohol vs. Cigarettes | -0.34 | 0.06 | -0.56 | -0.12 | -5.50 | < .001 |
| Alcohol vs. Cocaine | -0.21 | 0.06 | -0.43 | 0.01 | -3.34 | < .001 |
| Alcohol vs. Opioids | -0.47 | 0.06 | -0.68 | -0.25 | -7.48 | < .001 |
| Cigarettes vs. Cocaine | 0.13 | 0.06 | -0.08 | 0.35 | 2.16 | 0.050 |
| Cigarettes vs. Opioids | -0.12 | 0.06 | -0.34 | 0.09 | -1.98 | 0.077 |
| Cocaine vs. Opioids | -0.26 | 0.06 | -0.48 | -0.04 | -4.14 | < .001 |
| **Never** | | | | | | |
| Cannabis vs. Alcohol | -0.69 | 0.12 | -1.13 | -0.26 | -5.56 | < .001 |
| Cannabis vs. Cigarettes | -0.86 | 0.12 | -1.30 | -0.43 | -6.92 | < .001 |
| Cannabis vs. Cocaine | -0.80 | 0.12 | -1.24 | -0.36 | -6.43 | < .001 |
| Cannabis vs. Opioids | -0.85 | 0.12 | -1.28 | -0.41 | -6.80 | < .001 |
| Alcohol vs. Cigarettes | -0.17 | 0.12 | -0.60 | 0.27 | -1.36 | 0.231 |
| Alcohol vs. Cocaine | -0.11 | 0.12 | -0.54 | 0.33 | -0.87 | 0.447 |
| Alcohol vs. Opioids | -0.15 | 0.12 | -0.59 | 0.28 | -1.24 | 0.271 |
| Cigarettes vs. Cocaine | 0.06 | 0.12 | -0.37 | 0.50 | 0.49 | 0.686 |
| Cigarettes vs. Opioids | 0.02 | 0.12 | -0.42 | 0.45 | 0.12 | 0.937 |
| Cocaine vs. Opioids | -0.05 | 0.12 | -0.48 | 0.39 | -0.37 | 0.777 |
| A significant interaction between cannabis use status and the substance type (i.e., cannabis alcohol, cigarettes, cocaine, and opioids) F(1,8)=10.02, p<.001 was observed. Significant main effect differences were also found between cannabis use statuses, (F(1,2)=35.41, p<.001), and substance types (F(1,4)=240.49, p<.001). | | | | | | |

| **Table S4**. Post-hoc pairwise comparisons from perceived addictiveness and DSM-5 status multivariate mixed effects linear model | | | | | | |
| --- | --- | --- | --- | --- | --- | --- |
| Comparison | Estimate | SE | 95% CI Lower | 95% CI Upper | *t ratio* | *p value* |
| **Cannabis** |  |  |  |  |  |  |
| Never Used vs. None | 0.76 | 0.11 | 0.35 | 1.18 | 6.71 | < .001 |
| Never Used vs. Mild | 0.98 | 0.18 | 0.32 | 1.64 | 5.44 | < .001 |
| Never Used. vs. Moderate | 0.61 | 0.21 | -0.17 | 1.39 | 2.84 | 0.037 |
| Never Used vs. Severe | 0.55 | 0.17 | -0.07 | 1.17 | 3.25 | 0.010 |
| None vs. Mild | 0.22 | 0.15 | -0.34 | 0.78 | 1.44 | 0.602 |
| None vs. Moderate | -0.16 | 0.19 | -0.86 | 0.54 | -0.82 | 0.925 |
| None vs. Severe | -0.21 | 0.14 | -0.73 | 0.30 | -1.50 | 0.565 |
| Mild vs. Moderate | -0.38 | 0.24 | -1.24 | 0.49 | -1.59 | 0.502 |
| Mild vs. Severe | -0.43 | 0.20 | -1.16 | 0.29 | -2.17 | 0.190 |
| Moderate vs. Severe | -0.05 | 0.23 | -0.89 | 0.78 | -0.24 | 0.999 |
| **Alcohol** |  |  |  |  |  |  |
| Never Used vs. None | 0.04 | 0.11 | -0.35 | 0.43 | 0.39 | 0.995 |
| Never Used vs. Mild | 0.07 | 0.17 | -0.54 | 0.68 | 0.43 | 0.993 |
| Never Used. vs. Moderate | -0.04 | 0.21 | -0.80 | 0.72 | -0.19 | 1.000 |
| Never Used vs. Severe | -0.10 | 0.14 | -0.63 | 0.43 | -0.67 | 0.962 |
| None vs. Mild | 0.03 | 0.14 | -0.50 | 0.56 | 0.21 | 1.000 |
| None vs. Moderate | -0.08 | 0.19 | -0.78 | 0.62 | -0.42 | 0.993 |
| None vs. Severe | -0.14 | 0.12 | -0.58 | 0.30 | -1.16 | 0.774 |
| Mild vs. Moderate | -0.11 | 0.23 | -0.96 | 0.73 | -0.48 | 0.989 |
| Mild vs. Severe | -0.17 | 0.18 | -0.81 | 0.47 | -0.96 | 0.871 |
| Moderate vs. Severe | -0.06 | 0.22 | -0.85 | 0.73 | -0.27 | 0.999 |
| **Cigarettes** |  |  |  |  |  |  |
| Never Used vs. None | -0.10 | 0.10 | -0.45 | 0.26 | -1.01 | 0.852 |
| Never Used vs. Mild | -0.20 | 0.13 | -0.68 | 0.27 | -1.55 | 0.528 |
| Never Used. vs. Moderate | -0.10 | 0.13 | -0.58 | 0.37 | -0.78 | 0.936 |
| Never Used vs. Severe | -0.10 | 0.12 | -0.52 | 0.32 | -0.87 | 0.906 |
| None vs. Mild | -0.10 | 0.13 | -0.56 | 0.35 | -0.83 | 0.921 |
| None vs. Moderate | 0.00 | 0.13 | -0.46 | 0.45 | -0.03 | 1.000 |
| None vs. Severe | 0.00 | 0.11 | -0.41 | 0.40 | -0.03 | 1.000 |
| Mild vs. Moderate | 0.10 | 0.15 | -0.46 | 0.65 | 0.66 | 0.965 |
| Mild vs. Severe | 0.10 | 0.14 | -0.41 | 0.61 | 0.71 | 0.954 |
| Moderate vs. Severe | 0.00 | 0.14 | -0.51 | 0.51 | 0.00 | 1.000 |
| **Cocaine** |  |  |  |  |  |  |
| Never Used vs. None | 0.14 | 0.08 | -0.14 | 0.42 | 1.79 | 0.381 |
| Never Used vs. Mild | 0.07 | 0.30 | -1.02 | 1.17 | 0.24 | 0.999 |
| Never Used. vs. Moderate | -0.67 | 0.42 | -2.21 | 0.86 | -1.61 | 0.494 |
| Never Used vs. Severe | -0.05 | 0.16 | -0.64 | 0.54 | -0.33 | 0.998 |
| None vs. Mild | -0.07 | 0.30 | -1.16 | 1.03 | -0.23 | 0.999 |
| None vs. Moderate | -0.81 | 0.42 | -2.35 | 0.72 | -1.94 | 0.298 |
| None vs. Severe | -0.19 | 0.16 | -0.78 | 0.40 | -1.18 | 0.761 |
| Mild vs. Moderate | -0.74 | 0.51 | -2.61 | 1.12 | -1.46 | 0.588 |
| Mild vs. Severe | -0.12 | 0.33 | -1.33 | 1.09 | -0.37 | 0.996 |
| Moderate vs. Severe | 0.62 | 0.44 | -1.00 | 2.24 | 1.40 | 0.626 |
| **Never Used** |  |  |  |  |  |  |
| Cannabis vs. Alcohol | 0.63 | 0.14 | 0.12 | 1.13 | 4.56 | < .001 |
| Cannabis vs. Cigarettes | -0.84 | 0.12 | -1.29 | -0.40 | -6.90 | < .001 |
| Cannabis vs. Cocaine | -0.85 | 0.11 | -1.26 | -0.43 | -7.44 | < .001 |
| Alcohol vs. Cigarettes | -0.22 | 0.12 | -0.64 | 0.21 | -1.86 | 0.246 |
| Alcohol vs. Cocaine | -0.22 | 0.11 | -0.60 | 0.17 | -2.05 | 0.169 |
| Cigarettes vs. Cocaine | 0.00 | 0.09 | -0.31 | 0.32 | 0.02 | 1.000 |
| **No SUD** |  |  |  |  |  |  |
| Cannabis vs. Alcohol | 1.35 | 0.06 | 1.13 | 1.57 | 22.34 | < .001 |
| Cannabis vs. Cigarettes | -1.70 | 0.07 | -1.97 | -1.43 | -22.92 | < .001 |
| Cannabis vs. Cocaine | -1.47 | 0.07 | -1.71 | -1.22 | -22.10 | < .001 |
| Alcohol vs. Cigarettes | -0.35 | 0.08 | -0.63 | -0.08 | -4.70 | < .001 |
| Alcohol vs. Cocaine | -0.12 | 0.07 | -0.37 | 0.13 | -1.77 | 0.288 |
| Cigarettes vs. Cocaine | -0.23 | 0.08 | -0.53 | 0.06 | -2.91 | 0.019 |
| **Mild** |  |  |  |  |  |  |
| Cannabis vs. Alcohol | 1.54 | 0.20 | 0.82 | 2.25 | 7.87 | < .001 |
| Cannabis vs. Cigarettes | -2.03 | 0.18 | -2.69 | -1.37 | -11.21 | < .001 |
| Cannabis vs. Cocaine | -1.76 | 0.32 | -2.94 | -0.58 | -5.45 | < .001 |
| Alcohol vs. Cigarettes | -0.49 | 0.17 | -1.12 | 0.14 | -2.85 | 0.023 |
| Alcohol vs. Cocaine | -0.22 | 0.32 | -1.39 | 0.95 | -0.68 | 0.903 |
| Cigarettes vs. Cocaine | -0.27 | 0.31 | -1.41 | 0.87 | -0.87 | 0.823 |
| **Moderate** |  |  |  |  |  |  |
| Cannabis vs. Alcohol | 1.27 | 0.26 | 0.31 | 2.23 | 4.86 | < .001 |
| Cannabis vs. Cigarettes | -1.55 | 0.21 | -2.32 | -0.78 | -7.32 | < .001 |
| Cannabis vs. Cocaine | -2.12 | 0.46 | -3.79 | -0.46 | -4.66 | < .001 |
| Alcohol vs. Cigarettes | -0.28 | 0.21 | -1.06 | 0.50 | -1.31 | 0.559 |
| Alcohol vs. Cocaine | -0.85 | 0.45 | -2.50 | 0.80 | -1.88 | 0.235 |
| Cigarettes vs. Cocaine | 0.57 | 0.43 | -0.99 | 2.14 | 1.34 | 0.535 |
| **Severe** |  |  |  |  |  |  |
| Cannabis vs. Alcohol | 1.28 | 0.17 | 0.67 | 1.89 | 7.64 | < .001 |
| Cannabis vs. Cigarettes | -1.50 | 0.16 | -2.06 | -0.93 | -9.63 | < .001 |
| Cannabis vs. Cocaine | -1.45 | 0.20 | -2.17 | -0.72 | -7.30 | < .001 |
| Alcohol vs. Cigarettes | -0.22 | 0.14 | -0.72 | 0.28 | -1.60 | 0.377 |
| Alcohol vs. Cocaine | -0.17 | 0.18 | -0.85 | 0.50 | -0.94 | 0.785 |
| Cigarettes vs. Cocaine | -0.05 | 0.17 | -0.68 | 0.59 | -0.27 | 0.993 |
| A significant interaction between SUD severity for each substance and the perceived addictiveness of alcohol, cocaine, cannabis, and tobacco F(1,12)=4.13, p<.001. Significant main effect differences were found for substance type, F(1,3)=20.28, p<.001, but not severity, F(1,4)=0.41, p=.80. | | | | | | |

| **Table S5**. Number of participants in each DSM-5 category by substance type | | | | | |
| --- | --- | --- | --- | --- | --- |
|  | DSM-5 3-Month Category (Number of Symptoms) | | | | |
|  | Never Used | No SUD (0-1) | Mild (2-3) | Moderate (4-5) | Severe (6+) |
| Cannabis | 65 | 351 | 32 | 20 | 39 |
| Alcohol | 77 | 315 | 37 | 20 | 58 |
| Tobacco | 133 | 169 | 60 | 60 | 85 |
| Cocaine | 233 | 232 | 8 | 4 | 30 |
| Opioids | 226 | 234 | 13 | 2 | 32 |
